# Patients with radiographic axial spondylarthritis have an impaired dietary intake—a cross-sectional study with matched controls from northern Sweden

**DOI:** 10.1186/s13075-023-03126-3

**Published:** 2023-08-07

**Authors:** Erik Hulander, Tatiana Zverkova Sandström, Jeannette Beckman Rehnman, Lucy Law, Stefan Söderberg, Helena Forsblad-d’Elia

**Affiliations:** 1 Department of Rheumatology and Inflammation Research, Institute of Medicine, Sahlgrenska Academy, University of Gothenburg, PO Box 480, 405 30 Gothenburg, Sweden; 2 Department of Gastroenterology and Hepatology, Clinical Nutrition Unit, Sahlgrenska University Hospital, Region Västra Götaland, Gothenburg, Sweden; 3 Department of Nursing, Faculty of Medicine, Umeå University, Umeå, Sweden; 4 Department of Public Health and Clinical Medicine, Umeå University, Umeå, Sweden; 5 Department of Rheumatology, Sahlgrenska University Hospital, Region Västra Götaland, Gothenburg, Sweden

**Keywords:** Axial spondyloarthritis (D000089183), Ankylosing spondylitis (D013167), Diet (D004032), Malnutrition (D044342)

## Abstract

**Background:**

Radiographic axial spondyloarthritis (r-axSpA) is one of the most common chronic inflammatory rheumatic diseases, affecting about 0.2% of the Swedish population. Adequate nutritional intake is essential for maintaining physiological functions. A poor diet increases the risk of developing conditions such as obesity, osteoporosis, and/or atherosclerosis. Diet quality is also theorized to affect systemic inflammation. Dietary habits in patients with r-axSpA are largely unknown. The aims of this study were to assess dietary nutrient intake in r-axSpA patients and examine whether it differs compared to persons without r-axSpA.

**Methods:**

r-axSpA patients (modified NY criteria) at the rheumatology clinic in Region Västerbotten, northern Sweden, were invited to take part in the Backbone study which investigates disease severity and comorbidities. In total, 155 patients were included. Nutritional intake was assessed by the semi-quantitative food frequency questionnaire MiniMeal-Q. Controls were collected from the Swedish CArdioPulmonary bioImage Study (*n* = 30,154), a study that invited participants 50–64 years of age by random selection from the Swedish population register. Out of the 155 r-axSpA patients, 81 were in the same age span. Four controls were identified for each patient, matched on age (± 1 year), sex, and geographic location. Data on dietary intake was available for 319 controls. Statistical comparisons of dietary intake between patients with r-axSpA and controls were done by exact conditional logistic regression analysis, adjusted for country of birth, educational level, single household, weight, smoking status, and energy intake.

**Results:**

Patients had a comparatively significantly higher energy intake from carbohydrates, a lower fiber density, and a lower intake of marine omega-3 fatty acids. Furthermore, intake of vitamins D, E, and K as well as selenium, folate, calcium, magnesium, phosphorus, potassium, vitamin A, and β-carotene (a precursor of vitamin A and marker of vegetable and fruit intake) was significantly lower among patients compared to controls.

**Conclusions:**

Our results suggest that r-axSpA patients have an impaired dietary intake. Notably, intake was lower in several nutrients theorized to have anti-inflammatory properties (fiber density, marine-omega-3 fatty acids, vitamin D, and selenium). We further propose that nutrition screening might be incorporated into the management of r-axSpA patients.

**Supplementary Information:**

The online version contains supplementary material available at 10.1186/s13075-023-03126-3.

## Background

Radiographic axial spondyloarthritis (r-axSpA) is one of the most common chronic rheumatic inflammatory diseases. The prevalence of r-axSpA varies globally; in Europe, a prevalence of around 0.2% is assumed, affecting around one and a half million people in Europe alone [1]. Disease symptoms often begin early in life, leading to a relatively prolonged disease burden for afflicted individuals. Physiotherapy has proven benefits for patients with r-axSpA [2]. The implementation of biological disease-modifying anti-rheumatic drugs (bDMARDs) has also revolutionized the pharmacological treatment options, when non-steroidal anti-inflammatory drugs (NSAID) and physiotherapy are not sufficient. Nevertheless, maintained remission over time is uncommon [3], and a large proportion of patients with r-axSpA still experience residual pain, stiffness, a deteriorated physical capacity, and reduced health-related quality of life [4]. Patients with SpA themselves often experiment with different dietary regimens in the hope of alleviating their symptoms or to reach prolonged remission [5, 6].

A poor diet increases the risk of developing conditions such as obesity, atherosclerosis, diabetes, and/or osteoporosis. While obesity itself often leads to an altered immune function, dietary intake also correlates directly with inflammatory markers, thus several dietary inflammation indexes have been constructed [7–9]. In general, whole grains and plant-based foods are categorized as anti-inflammatory, while highly processed and sugar-sweetened foods are considered pro-inflammatory. Nutritional shortages may further influence the immune system. For instance, vitamin D is important for a wide range of immunologic processes [10]. An adequate intake of dietary fiber supports the intestinal barrier by promoting a commensal micro-biotic culture and the production of short-chain fatty acids [11]. Likewise, an adequate intake of omega-3 fatty acids modulates the cyclooxygenase and 5-lipoxygenase pathways to the production of less inflammatory eicosanoids [12].

When it comes to evidence on dietary treatment of patients with r-axSpA, there is no guideline on what diet to recommend [13, 14], reflecting the scarce evidence. In fact, only a handful of dietary intervention studies on patients with r-axSpA have been performed. They have focused on topics such as a reduced starch intake [15] or a milk-free diet [16]. However, the lack of randomized control groups hinders a definitive conclusive interpretation of these studies. Intervention studies on dietary supplements have so far mostly led to null findings [17–19] or have had a study design without a control group, which again renders a causal interpretation problematic [20]. The protocol of an ongoing randomized controlled trial (RCT) on a gluten-free diet has been published, but results are not yet available [21].

Observational studies indicate that the intestinal microbiota differs between patients with r-axSpA compared to controls [22–24] and that the biota is related to disease activity and dietary intake [22]. Dietary intake patterns appear to differ between patients with r-axSpA and controls [22]. Within patients with r-axSpA, ultra-processed food intake appear to correlate with higher disease activity [25], while intake of marine omega-3 fatty acids appears to correlate with lower inflammation [26]. Altogether, there are indications that diet might affect disease outcomes in patients with r-axSpA. However, as of today, data on nutritional intake in patients with r-axSpA compared to the general population has been poorly described. The aim of this study is, therefore, to assess nutritional intake in patients with r-axSpA, and whether it differs between patients with r-axSpA and age- and sex-matched controls.

## Methods

### Patients and controls

This study is based on data obtained from a cohort study, called the Backbone study. This cohort has been described in detail previously [27, 28]. In brief, all patients with a diagnosis of r-axSpA according to the modified New York criteria [29], listed at the rheumatology clinic in Region Västerbotten in Northern Sweden, 18–75 years of age, were invited to take part. The exclusion criteria were pregnancy, difficulties in understanding the Swedish language, any other inflammatory diseases, and dementia. In total, 155 patients were included. Blood samples were collected, and C-reactive protein (CRP) and erythrocyte sedimentation rate (ESR) were analyzed by standard clinical laboratory routines. Measures of disease activity, physical function, back mobility, and r-axSpA-related radiographic spinal alterations were assessed with the Ankylosing Spondylitis Disease Activity Score with CRP (ASDAS-CRP), the Bath Ankylosing Disease Spondylitis Activity Index (BASDAI), the Bath Ankylosing Spondylitis Functional Index (BASFI), the Bath Ankylosing Spondylitis Metrology Index (BASMI), and the Modified Stoke Ankylosing Spondylitis Spinal Score (mSASSS), respectively [30].

For the recruitment of controls without r-axSpA, data from the Swedish CArdioPulmonary bioImage Study (SCAPIS) cohort was used. The SCAPIS study has been described in detail elsewhere [31]. In brief, this is a national effort to gather information on the development of cardiovascular and obstructive pulmonary diseases and related metabolic disorders in Sweden. This study included 30,154 subjects between the ages 50–64 years randomly selected from the Swedish population register in six geographical areas. The only exclusion criterion was the inability to sign the informed consent agreement due to difficulties in understanding written or spoken Swedish. At the Umeå University Hospital in northern Sweden, 2506 participants were included.

From the SCAPIS cohort in Umeå, participants who had answered no to having any rheumatic inflammatory disease, matched on sex and age (± 1 year), were included as controls. For each patient, four controls were selected. In total, 324 controls were matched to 81 patients, 49–65 years old.

Both patients and controls answered questionnaires on the place of birth, living conditions, smoking habits, and educational level. Weight was measured to the closest hectogram and height to the closest centimeter.

### Assessing nutritional intake

Habitual nutritional intake was assessed by the semi-quantitative food frequency questionnaire (FFQ) *MiniMeal-Q.* As previously described elsewhere [32, 33], this questionnaire is developed for a Swedish setting and is designed to be functional as a self-administered interactive test. This questionnaire is a meal-based FFQ; it asks for a range of commonly consumed types of meals, and only if consumed frequently enough, the specific types and amounts of foods in the meal are asked for in detail. Thus, the questionnaire includes between 75 and 126 food items and in theory compiles a list of foods and amounts with the highest impact on the individual’s diet. After this, macro- and micronutrient data are calculated using the food database supplied by the Swedish Food Agency. The same questionnaire was used for assessing nutritional intake in both the SCAPIS and the Backbone study; in the Backbone study, the patients filled it out on paper, and in the SCAPIS study, it was filled out electronically. When comparing nutritional intake to recommendations, the Nordic Nutrition Recommendations was used as a reference [34].

### Statistical analysis

For hypothesis tests of continuous variables, *t*-tests are presented. Non-normally distributed variables were transformed by Log_10_ or Log_e_, and if still not normally distributed, a Mann–Whitney test with the exact *p*-value was used. For categorical data, Fisher’s exact test was applied.

An exact logistic regression was used to assess the differences in nutritional intake between patients and controls. Smoking ever (previous or ongoing) status (yes/no), country of birth (Sweden or abroad), and cohabiting status (single household or not) were included as dichotomous variables. Energy intake and weight were included as continuous variables, and educational level was included as a categorical variable (1, junior high school or missing; 2, senior high school; 3, university or college). One patient and one control lacked data on educational level.

For the exact conditional logistic regression model, SAS version 9.4 was utilized. For all other analyses, IBM SPSS version 29 was used. For graphical presentation, RStudio 2022.12.0 + 353 was used. No correction for multiple hypothesis tests was applied. The significance level was assumed at *p*-values < 0.05.

For a sensitivity analysis, participants with the lowest and highest energy intakes were excluded from the analysis. This was done in two ways: first, the method previously applied by Bälter et al. [35], removing those with a daily energy intake > 21,000 or < 3300 kJ; second, the method applied by Sundström et al. [36], who divided daily energy intake by basal metabolic rate (BMR) (estimated by the Schofield equation [37]), removing participants < 5th or > 97.5th percentile.

## Results

**Fig. 1:**
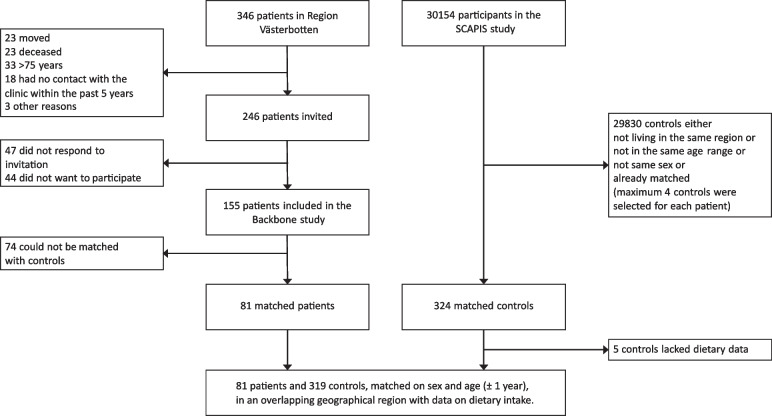
Inclusion of patients with r-axSpA from the Backbone study and controls from the SCAPIS study

Out of the 155 patients in the Backbone study, 81 patients were in the same age span as participants in SCAPIS and could be matched with controls (Fig. 1). Nutritional intake was available from 319 age- and sex-matched controls (Table 1). Patients with r-axSpA were shorter than the controls and had a higher body mass index (BMI), but no other demographical variables differed.

**Table 1:** Characteristics of patients with r-axSpA and matched controls

	Patients (*n* = 81), mean ± SD or *N* (%)	Controls (*n* = 319), mean ± SD or *N* (%)	*p*-value
Age (years)	57.9 ± 4.8	58 ± 4.6	1.0^a^
Male	54 (66.7)	211 (66.1)	1.0^b^
Born in Sweden	79 (97.5)	296 (92.8)	0.2^b^
BMI (kg/m^2^)	28.7 ± 6.0	27.1 ± 4.6	**0.027**^**a**^
Height (cm)	171.5 ± 9.6	174.8 ± 8.5	**0.003**^**a**^
Weight (kg)	84.9 ± 21.8	82.9 ± 15.8	0.4^a^
HLA-B27 positive	80 (98.8)		
Age at diagnosis (years)	32.8 ± 8.6	–	
Symptom duration (years)	34.6 ± 8.4	–	
Diagnosis duration (years)	25.1 ± 9.6	–	
ASDAS-CRP (score)	1.9 ± 0.8	–	
BASDAI (score)	4.0 ± 1.9	–	
BASFI (score)	3.3 ± 2.2	–	
BASMI (score)	4.4 ± 1.4	–	
mSASSS^c^ (score)	22.3 ± 22.2	–	
Median (25p, 75p) (score)	16.0 (3.3, 36.3)	–	
Anti-rheumatic medication			
csDMARD and/or bDMARD	19 (23.5)	–	
csDMARD	9 (11.1)	–	
bDMARD	15 (18.5)	–	
NSAID use frequency			
None	13 (16.0)	–	–
On demand	19 (23.5)	–	–
≥ 2 days/week	49 (60.5)	–	–
Folate supplementation			
Self-reported use	9 (11.1)	–	
Prescribed by a physician	7 (8.6)	–	
Prescribed or self-reported	12 (14.8)	–	
Educational level^d^			0.2^b^
Junior high school or lower	6 (7.4)	35 (11.0)	
Senior high school	51 (62.9)	167 (52.4)	
College or university	24 (29.6)	117 (36.7)	
Single household	18 (22.2)	51 (16.0)	0.2^b^
Ever smoker^e^	42 (51.8)	143 (44.8)	0.3^b^

*Abbreviations*: *ASDAS-CRP *Ankylosing Spondylitis Disease Activity Score CRP, *BASDAI *Bath Ankylosing Spondylitis Disease Activity Index, *BASFI *Bath Ankylosing Spondylitis Functional Index, *BASMI* Bath Ankylosing Spondylitis Metrology Index, *bDMARD *biologic disease-modifying anti-rheumatic drug, *csDMARD *conventional synthetic disease-modifying anti-rheumatic drug, *HLA-B27 *human leukocyte antigen B27, *mSASSS *Modified Stoke Ankylosing Spondylitis Spinal Score, *NSAID *non-steroidal anti-inflammatory drug, *r-axSpA *radiographic axial spondyloarthritis

^﻿a^Mann Whitney test, using exact *p*-value or *T*-test depending on data distribution

^b^Fisher’s exact test

^﻿c^Data missing from one individual, due to non-normal distribution median and 25th and 75th percentiles are also shown

^d^Data missing from one patient with r-axSpA and one control, categorized as junior high school or lower

^e^Data missing from five in the control group

### Macronutrients

In a comparison of mean intakes, patients had a higher energy percentage (E%) from carbohydrates and a lower fiber density than controls (Table 2). Furthermore, the patients had a comparatively lower intake of marine omega-3 fatty acids. The E% of omega-6 fatty acids, essential fatty acids, and linoleic acid was comparatively also lower among patients.

**Table 2:** Macronutrient and energy intake in patients with r-axSpA compared with matched controls

	Patients (*n* = 81), mean ± SD	Controls (*n* = 319), mean ± SD	Difference (95% CI)	Recommended intake^a^
Energy, kcal	1 810 ± 727	1 740 ± 652	70 (− 104 to 244)	–
Carbohydrates, g	203 ± 88	187 ± 81	16 (− 6 to 37)	–
Fiber, g	19.6 ± 9.25	20.2 ± 10	− 0.6 (− 2.9 to 1.7)	≥ 25/35^c^
Fiber, g/MJ	2.56 ± 0.68	2.80 ± 0.96	− 0.24 (− 0.42 to − 0.06)	≥ 3
Alcohol^b^, g	6.24 ± 5.02	7.40 ± 6.47	− 1.16 (− 2.47 to 0.15)	≤ 10/20^c^
Protein, g	70.4 ± 29	68.0 ± 24.6	2.4 (− 4.5 to 9.3)	–
Protein, g/kg	0.87 ± 0.39	0.84 ± 0.34	0.03 (− 0.06 to 0.12)	0.8–1.5
Total fat, g	70.2 ± 31.3	70.2 ± 29.1	0.0 (− 7.5 to 7.6)	–
Marine omega-3 fatty acids^d^, mg	354 ± 265	438 ± 336	− 84 (− 152 to − 15)	–
EPA, mg	78 ± 62.2	123 ± 103	− 45 (− 63 to − 27)	–
DHA, mg	245 ± 179	256 ± 195	− 10 (− 55 to 34)	–
DPA, mg	31 ± 26	59 ± 40	− 28 (− 35 to − 21)	–
**Energy percentages**				
Carbohydrates, E%	44.9 ± 6.2	43.4 ± 6.8	1.59 (0.04 to 3.14)	45–60
Alcohol^b^, E%	2.75 ± 2.81	3.18 ± 2.75	− 0.44 (− 1.12 to 0.25)	≤ 5
Protein, E%	16.0 ± 2.7	16.2 ± 2.9	− 0.1 (− 0.8 to 0.5)	10/15–20^e^
Total fat, E%	34.2 ± 4.94	35.5 ± 5.45	− 1.26 (− 2.49 to − 0.03)	25–40
Saturated fatty acids, E%	13.8 ± 2.8	13.9 ± 3.0	− 0.1 (− 0.8 to 0.6)	< 10
Trans fatty acids, E%	0.37 ± 0.16	0.34 ± 0.15	0.03 (− 0.01 to 0.07)	
Monounsaturated fatty acids, E%	12.5 ± 1.99	13.0 ± 2.43	− 0.47 (− 0.98 to 0.04)	10–20
Polyunsaturated fatty acids, E%	5.19 ± 1.34	5.70 ± 2.21	− 0.51 (− 0.89 to − 0.13)	5–10
Omega-6 fatty acids^f^, E%	3.86 ± 1.03	4.31 ± 1.75	− 0.44 (− 0.74 to − 0.15)	–
Omega-3 fatty acids^g^, E%	1.12 ± 0.569	1.21 ± 0.68	− 0.09 (− 0.24 to 0.05)	≥ 1
Essential fatty acids^h^, E%	4.77 ± 1.32	5.23 ± 2.14	− 0.46 (− 0.83 to − 0.09)	≥ 3
Linoleic acid, E%	3.83 ± 1.02	4.26 ± 1.74	− 0.43 (− 0.72 to − 0.13)	–
Alpha linolenic acid, E%	0.94 ± 0.57	0.97 ± 0.64	− 0.04 (− 0.18 to 0.11)	≥ 0.5
Marine omega-3 fatty acids^d^, E%	0.18 ± 0.13	0.24 ± 0.18	− 0.06 (− 0.09 to − 0.02)	–

*Abbreviations*: *DHA *docosahexaenoic acid, *DPA *docosapentaenoic acid, *EPA *eicosapentaenoic acid, *MJ *megajoule, *r-axSpA *radiographic axial spondyloarthritis

^a^As outlined in the Nordic Nutrition Recommendations [34]

^b^Data missing from 12 controls

^c^Lower value valid for females, higher value for males

^d^DHA, DPA, and EPA combined

^e^ ≥ 65 year olds: 15–20 E%; ˂ 65 year olds: 10–20 E%

^f^The sum of arachidonic acid and linoleic acid

^g^The sum of alpha linoleic acid, EPA, DHA, and DPA

^h^The sum of alpha linoleic acid and linoleic acid

**Fig. 2:**
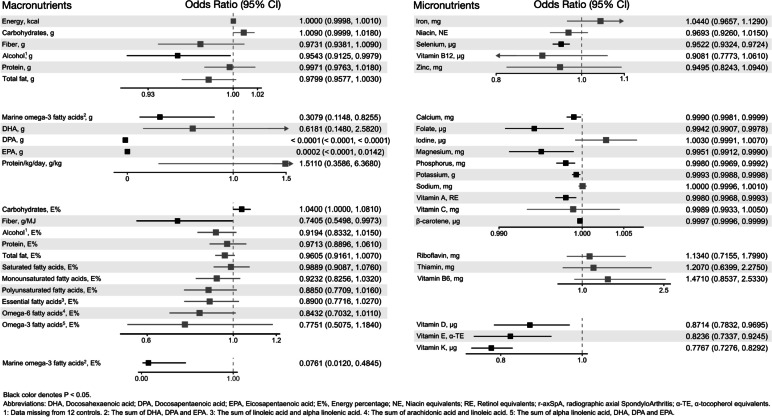
Nutrient intakes in patients with r-axSpA compared to controls with an exact conditional logistic regression

In the regression models, patients with r-axSpA had a higher E% intake from carbohydrates and a lower fiber density (Fig. 2, Additional file 1: Table S1). Additionally, intakes of marine omega-3 fatty acids and alcohol (in grams but not E%) were lower in patients with r-axSpA compared to controls.

### Micronutrients

When comparing the mean intakes, patients had lower intakes of magnesium, selenium, vitamins E and K, and β-carotene (Table 3).

**Table 3:** Daily micronutrient intake in patients with r-axSpA compared with matched controls

	Patients (*n* = 81), mean ± SD	Controls (*n* = 319), mean ± SD	Difference (95% CI)	Recommended intake^a^
Calcium, mg	831 ± 432	875 ± 450	− 44 (− 150 to 62)	800
Folate, µg	264 ± 117	290 ± 117	− 26 (− 54 to 3)	300/400^b^
Iodine, µg	128 ± 88	109 ± 69	19 (− 1 to 40)	150
Iron, mg	11.5 ± 5.6	10.7 ± 4.8	0.8 (− 0.5 to 2.2)	9/15^b^
Magnesium, mg	331 ± 139	342 ± 126	− 11 (− 45 to 22)	280^c^/350^d^
Niacin, NE	30.4 ± 12.1	30.3 ± 9.7	0.1 (− 2.7 to 3.0)	14^c^^,e^/13^c,f^/18^d,e^/16^d,f^
Phosphorus, mg	1280 ± 562	1330 ± 523	− 50 (− 185 to 85.8)	600
Potassium, mg	2850 ± 1120	2960 ± 910	− 110 (− 360 to 160)	3100^c^/3500^d^
Riboflavin, mg	1.59 ± 0.80	1.48 ± 0.75	0.11 (− 0.08 to 0.31)	1.2^c^/1.5^d,e^/1.4^d,f^
Selenium, µg	35.1 ± 16.2	46.0 ± 25.6	− 10.9 (− 15.4 to − 6.38)	50^c^/60^d^
Sodium, mg	2500 ± 1070	2 390 ± 909	110 (− 145 to 361)	< 2400 mg
Thiamin, mg	1.30 ± 0.61	1.22 ± 0.54	0.08 (− 0.06 to 0.23)	1.1^c,e^/1.0^c,f^/1.3^d,e^/1.2^d,f^
Vitamin A, RE	652 ± 297	725 ± 301	− 73 (− 145 to 0.178)	700^c^/900^d^
Vitamin B12, µg	4.17 ± 2.01	4.20 ± 2.11	− 0.03 (− 0.53 to 0.46)	2.0
Vitamin B6, mg	1.77 ± 0.78	1.64 ± 0.63	0.13 (− 0.06 to 0.31)	1.2^c,e^/1.3^c,f^/1.5^d^
Vitamin C, mg	72.1 ± 58.6	74.1 ± 43.8	− 2.0 (− 15.6 to 11.7)	75
Vitamin D, µg	5.98 ± 3.01	6.46 ± 3.47	− 0.48 (− 1.24 to 0.27)	10
Vitamin E, µg α-TE	8.26 ± 3.81	9.23 ± 3.77	− 0.97 (− 1.9 to − 0.04)	8^c^/10^d^
Vitamin K, µg	13.8 ± 8.06	27.6 ± 14.5	− 13.8 (− 16.2 to − 11.4)	1 µg/kg
Zinc, mg	9.72 ± 4.04	9.49 ± 3.58	0.23 (− 0.73 to 1.2)	7^c^/9^d^
β-Carotene, µg	2520 ± 1740	3700 ± 2470	− 1180 (− 1650 to − 714)	–

*Abbreviations*: *RE *retinol equivalents, *r-axSpA *radiographic axial spondyloarthritis, *α-TE *alpha-tocopherol equivalents, *r-axSpA *radiographic axial spondyloarthritis

^a^As outlined in the Nordic Nutrition Recommendations [34]

^b^Females of reproductive age

^c^Females

^d^Males

^e^31–60 years of age

^f^61–74 years of age

The regression model indicated that patients with r-axSpA had a lower intake of folate and vitamins A, D, E, and K (Fig. 2, Additional file 1: Table S1). The patients also had a lower intake of calcium, magnesium, phosphorus, potassium, and selenium. Additionally, the intake of β-carotene was markedly lower in patients compared to controls.

### Adherence to recommended daily intakes of micronutrients

**Fig. 3:**
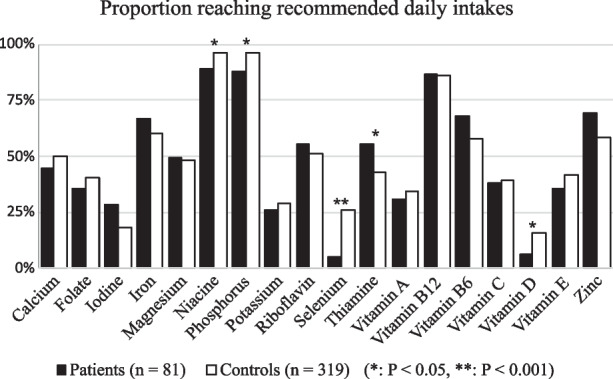
Proportion of patients with radiographic axial spondyloarthritis and controls reaching recommended daily intakes of micronutrients

In comparison with controls, a lower proportion of patients with r-axSpA met the recommended daily intake (RDI)s of niacin, phosphorus, selenium, and vitamin D (Fig. 3). Only the RDI of thiamin was met by a higher proportion of patients with r-axSpA compared to controls.

### Comparisons between patients in Backbone included and not included in this investigation

Controls from SCAPIS were not available in all ages, and thus, about half of the patients were excluded from analyses (43 patients were below 49 years of age, and 31 were above 66 years of age). The characteristics of patients with r-axSpA included and excluded from comparison to controls differed in some characteristics (Additional file 1: Table S2). While the median ages did not differ, the age range was wider in those excluded. Furthermore, the mSASSS was higher, and the educational level was lower among those excluded.

### Sensitivity analysis

Both sensitivity analyses gave largely similar results as the main analyses; below are the results that were altered. When including only participants with a daily energy intake between 3300 and 21,000 kJ, 75 patients with r-axSpA and 287 matched controls remained. Alcohol intake in E% became significantly lower, but alcohol intake in grams was no longer lower in patients compared to controls (*p* = 0.085). Absolute intake of marine omega-3 fatty acids no longer differed (*p* = 0.066) between patients and controls, but intake of eicosapentaenoic acid (EPA) and docosapentaenoic acid (DPA) was still significantly lower in patients. Moreover, the intake of trans fatty acids in E% was significantly higher in patients compared to controls, and the E% of omega-6 fatty acids was comparatively lower.

When including only participants within the 5th and 97.5th percentile range of energy intake divided by BMR, 71 patients with r-axSpA and 263 matched controls remained. This analysis indicated significantly higher energy and carbohydrate intake in patients compared to controls and lower fiber intake. Furthermore, intake of calcium and vitamin D no longer differed between patients and controls (*p* = 0.081 and *p* = 0.060, respectively). Intake of alcohol in E% was significantly lower, E% of trans fatty acids was higher, and E% of omega-6 fatty acids was lower in patients compared to controls.

## Discussion

This study explored whether the nutritional intake differed between patients with r-axSpA compared to a control group matched on age and sex living in the same region in Sweden. Our main findings are that dietary nutrient intake is impaired in patients with r-axSpA; diet quality is lower than among persons without r-axSpA.

Our main analysis was done with a conditional logistic regression model, analyzing the differences within the strata of patients and matched controls. This analysis was adjusted for factors that we hypothesized could affect dietary intake. Dietary habits can be intricately linked to social interactions, and we therefore adjusted for household status (living alone or not). Education level and smoking status were included as socioeconomic markers. Since the FFQ was developed for foods commonly consumed in Sweden, and might not capture the dietary intake as well in foreign populations, country of birth (Sweden or abroad) was included as a marker of Swedish food culture. Since obese participants often more heavily underreport their dietary intake [38], and since adjustment for BMI would introduce a bias between patients and control, weight was included as a covariate. Lastly, energy intake, as an ancestral variable to all nutrient intakes, was also adjusted for.

The results from our main analysis indicate a lower intake of marine omega-3 fatty acids, higher intake of carbohydrates and lower fiber density, a lower intake of vitamins A, D, E, and K as well as calcium, folate, phosphorus, potassium, selenium, and β-carotene.

### Potential impact of our findings on the patient population

Selenium, mainly found in marine foods and offal foods, is a cofactor to several enzymes, and its role in the antioxidative glutathione peroxidases and its importance for the immune system have been described [39]. As documented in a recent systematic review [40], low levels of selenium have been found in populations with other rheumatic diseases, and although not proven in intervention studies, the authors propose that an optimal selenium status might improve disease outcomes.

The influence of marine-omega-3 fatty acids in general, and EPA in particular, on the immune system through providing less inflammatory eicosanoids in the cyclooxygenase and 5-lipoxygenase pathways is a well-established theory [12]. To the best of our knowledge, there has been only one intervention trial on patients with r-axSpA with fish oil, this trial compared two different dosages but saw no significant difference [19]. The effect of intervention with fish oils is better studied in patients with RA, where a reduction in leukotrienes as well as less pain and stiffness has been found [41].

Potassium, folate, and vitamin K are all nutrients that, similar to β-carotene, are found mainly in vegetable food sources. In general, an increased intake of potassium may lower blood pressure and thus prevent cardiovascular events [42]. In patients with RA, one RCT found that supplemental potassium in those deficient had beneficial effects on disease activity [43]. Folate is perhaps most known for its function as a nucleic acid precursor in cell divisions. Deficiency is seen first in tissues with a high turnover rate, and a common sign is megaloblastic anemia. Vitamin K is essential for blood coagulation, foods with the highest concentration are generally dark green vegetables. However, vitamin K is also produced by the intestinal microbiota and deficiency in humans with an intact intestinal function has not been described [34].

Vitamins A, D, and E are fat-soluble nutrients. Vitamin E is mainly found in plant seeds and oils and has antioxidative functions. Vitamin A is mainly found in liver, marine, and dairy and as carotenoid precursors in vegetable foods and is essential for maintaining normal eyesight.

The primary function of vitamin D, a semi-hormone that can also be produced endogenously by sun exposure on the human skin, is to regulate calcium and phosphorus uptake and metabolism. Deficiency can result in impaired bone development and contribute to the development of osteoporosis. Vitamin D status has been described to inversely correlate with inflammatory disease but is also an acute phase reactant that drops when tissue injury occurs. Thus, it has been unclear whether a lower vitamin D status in patients with inflammatory diseases, such as has been described in patients r-axSpA [44], is a causal factor or merely negatively correlated to inflammation for other reasons. Recently, results from a large-scale RCT study (*n* = 25.871) on vitamin D supplementation found a decreased incidence of autoimmune diseases [45] and indications of reduced CRP after 2 but not 4 years [46]. Thus, there appears to be some substance to the theory that an adequate vitamin D status can be beneficial for patients with inflammatory diseases.

Phosphorus is found in a wide range of foods of both animal and plant origin and has numerous functions in the human body. For example, it is essential for the structure of cell membranes, bone mineralization, and energy metabolism. Deficiency is very rare in Sweden but may lead to osteoporosis in the long term, and the current recommended intake is based upon maintaining an equimolar ratio between the intake of phosphorus and calcium [34]. Calcium is an essential nutrient important for functions, such as cell signal transductions and enzymatic reactions. However, it is tightly regulated in the circulation, and the largest deposition is in the teeth and bones. Chronic low intake of calcium may thus lead to the development of osteoporosis. In Sweden, dairy products are the main source of calcium although marine products and several other foods contribute [34]. While fewer of the patients met the RDI of phosphorus compared to controls (Fig. 3), the mean daily intake of phosphorus and calcium was above the RDI (Table 3). Magnesium is another mineral where deficiency is uncommon. It is essential for the metabolism of protein and calcium and is mainly found in legumes, vegetables, meat, and marine foods. The clinical relevance of the lower intake of calcium, phosphorus, and magnesium in patients compared to controls is not clear but is nevertheless indicative of a low nutrient density diet.

### Other dietary studies

Similar to what has been found in the general population in Sweden [47], both patients and the controls in our study had an unfavorably high intake of saturated fatty acids and a lower-than-optimal fiber intake. In contrast, however, both our patients and controls appear to cover recommended daily intakes more poorly compared to the results of the latest national food survey [47]. However, this survey utilized a different dietary assessment method, in a wider age range (18–80 years) and with a response rate of 36%. Furthermore, it was done over a decade ago and dietary trends could have changed slightly since then.

Data on dietary nutrient intake in patients with r-axSpA is rather scarce and to the best of our knowledge, only one similar study has been performed. Sundström et al. found a higher energy intake in patients with r-axSpA compared to controls [36], but no other differences. Similar to our analysis, Sundström et al. applied a conditional logistic regression model, but no additional variables were adjusted for. In that study, controls were recruited from a different cohort and in a slightly different age spectrum, and the FFQ had fewer questions. The differences in dietary assessment method, the matching procedure of controls, and the statistical analysis could be some explanatory factors to the divergent results.

To this date, no RCT diet study has been published on patients with r-axSpA. Previous dietary interventions in patients with rheumatoid arthritis (RA) indicate that dietary interventions such as the Mediterranean diet, i.e., an increased intake of foods that our patients appear to have a lower intake of, might affect systemic inflammation and disease activity [48]. These dietary interventions have often led to concurrent weight loss. However, beneficial effects have recently been described also in weight-stable patients with RA [49, 50]. It remains to be determined if this type of dietary intervention could be beneficial for patients with r-axSpA.

### Limitations

Our study design, with a cross-sectional analysis, does not allow for causal interpretations to be drawn. Furthermore, our data are limited to those with rather long-standing disease duration. It would be interesting to study also the newly diagnosed patients. While an impaired dietary intake could hypothetically affect the disease course, it is equally possible that patients with r-axSpA uphold a poorer dietary intake than the controls due to fatigue, or a stronger preference for comfort foods. It is also possible, since patients filled out the questionnaire on paper, and controls electronically, that we have introduced a bias. The more agile participants who are able to fill out electronic questionnaires could hypothetically have a higher socioeconomic status.

Furthermore, our results are limited to the age span of around 50–65 years of age, the findings are not necessarily valid in patient groups of other ages or those residing in other geographical regions. Additionally, the dietary data does not detail the actual foods consumed, but rather only the nutrient intakes. Thus, we cannot determine how the intake of specific foods differed between patients and controls.

Lastly, another limitation of our study is the number of hypothesis tests; the results are prone to type 1 errors. However, virtually, all results indicate uniformly that patients with r-axSpA have a lower dietary quality than the controls. Only one micronutrient in one analysis was higher in patients with r-axSpA compared to controls: the proportion reaching RDI of thiamin (Fig. 3). Thiamine is necessary for the metabolism of carbohydrates and branched-chain amino acids [34], and the main dietary sources of thiamin in the Swedish population has previously been attributed to cereal, meat, and dairy products [47]. In Sweden, thiamin deficiency is rare in those with a functional gastrointestinal tract, but it can be found in individuals with alcohol abuse.

### Strengths

There are several strengths in this investigation that merit mentioning. We utilized a control population from a cohort that aimed at recruiting a population representative to the general population. The SCAPIS study invited randomly selected participants from the population register, whom we matched tightly by age and sex to our patients. Furthermore, we used the same tool for collecting data on nutritional intake, in patients as well as in controls, thereby reducing bias in reporting. We utilized a regression model adjusted for potentially important confounders in our main analysis, and we tried two sensitivity analyses that gave similar results. There is likely always a selection bias in those who participate in clinical studies; still, both the patients with r-axSpA and the controls were recruited systematically in overlapping geographic locations, thereby minimizing the risk of a systemic recruitment bias.

## Conclusion

Our results indicate that patients with r-axSpA have an impaired dietary intake, compared to matched controls recruited randomly from the general population without a rheumatic diagnosis. Our study design does not allow to draw conclusions on whether dietary impairment exacerbates disease severity, or whether a reduced diet quality is secondary to the disease course. The nutritional impact on disease outcomes in patients with r-axSpA merits further investigation in different age ranges and geographic locations. The clinical care of patients with r-axSpA might be improved by the incorporation of nutrition screening.

### Supplementary Information

**Additional file 1:** **Table S1.** Output from the exact conditional logistic regression model comparing patients with r-axSpA to matched controls. **Table S2.** Patients with r-axSpA included in the comparison to controls, and patients not included.

## Data Availability

The datasets generated and/or analyzed during the current study are not publicly available due to Swedish privacy laws but are available from the corresponding author upon reasonable request.

## Abbreviations

ASDAS-CRP: Ankylosing Spondylitis Disease Activity Score CRP
BASDAI: Bath Ankylosing Spondylitis Disease Activity Index
BASFI: Bath Ankylosing Spondylitis Functional Index
BASMI: Bath Ankylosing Spondylitis Metrology Index
bDMARD: Biologic disease-modifying anti-rheumatic drug
BMI: Body mass index
BMR: Basal metabolic rate
CRP: C-reactive protein
csDMARD: Conventional synthetic disease-modifying anti-rheumatic drug
DPA: Docosapentaenoic acid
E%: Energy percentage
EPA: Eicosapentaenoic acid
ESR: Erythrocyte sedimentation rate
FFQ: Food frequency questionnaire
mSASSS: Modified Stoke Ankylosing Spondylitis Spinal Score
NSAID: Non-steroidal anti-inflammatory drug
RA: Rheumatoid arthritis
r-axSpA: Radiographic axial spondyloarthritis
RCT: Randomized controlled trial
RDI: Recommended daily intake
SCAPIS: Swedish CArdioPulmonary bioImage Study
