# Supplementary material for: Patients with radiographic axial spondylarthritis have an impaired dietary intake—a cross-sectional study with matched controls from northern Sweden
Source: Arthritis Res Ther. 2023 Aug 7;25:142. doi: 10.1186/s13075-023-03126-3 (PMC10405516; doi:10.1186/s13075-023-03126-3)
Supplement: Supplementary file 1 — Additional file 1: Table S1. Output from the exact conditional logistic regression model comparing patients with r-axSpA to matched controls. Table S2. Patients with r-axSpA included in the comparison to controls, and patients not included. [file 13075_2023_3126_MOESM1_ESM.docx]

**Supplemental Table 1**. Output from the exact conditional logistic regression model comparing patients with r-axSpA to matched controls^1^

|  | Odds Ratio (95% CI) | P-value |
| --- | --- | --- |
| Macro nutrients and energy intake |  |  |
| Energy, kcal | 1.0000 (0.9998, 1.0010) | 0.4125 |
| Carbohydrates, g | 1.0090 (0.9999, 1.0180) | 0.0540 |
| Fiber, g | 0.9731 (0.9381, 1.0090) | 0.1432 |
| Fiber, g/MJ | 0.7405 (0.5498, 0.9973) | **0.0480** |
| Alcohol^2^, g | 0.9543 (0.9125, 0.9979) | **0.0404** |
| Protein, g | 0.9971 (0.9763, 1.0180) | 0.7852 |
| Protein, g/kg | 1.5110 (0.3586, 6.3680) | 0.5737 |
| Total fat, g | 0.9799 (0.9577, 1.0030) | 0.0811 |
| Marine omega-3 fatty acids^3^, g | 0.3079 (0.1148, 0.8255) | **0.0192** |
| EPA, g | 0.0002 (< 0.0001, 0.0142) | **0.0001** |
| DHA, g | 0.6181 (0.1480, 2.5820) | 0.5095 |
| DPA, g | < 0.0001 (< 0.0001, < 0.0001) | **< 0.0001** |
|  |  |  |
| Proportion of energy intake | |  |
| Carbohydrates, E% | 1.0400 (1.0000, 1.0810) | **0.0490** |
| Alcohol^2^, E% | 0.9194 (0.8332, 1.0150) | 0.0943 |
| Protein, E% | 0.9713 (0.8896, 1.0610) | 0.5162 |
| Total fat, E% | 0.9605 (0.9161, 1.0070) | 0.0957 |
| Trans fatty acids, E% | 4.3550 (0.9075, 20.9000) | 0.0660 |
| Saturated fatty acids, E% | 0.9889 (0.9087, 1.0760) | 0.7952 |
| Monounsaturated fatty acids, E% | 0.9232 (0.8256, 1.0320) | 0.1607 |
| Polyunsaturated fatty acids, E% | 0.8850 (0.7709, 1.0160) | 0.0826 |
| Essential fatty acids^4^, E% | 0.8900 (0.7716, 1.0270) | 0.1095 |
| Omega-6 fatty acids^5^, E% | 0.8432 (0.7032, 1.0110) | 0.0654 |
| Linoleic acid, E% | 0.8492 (0.7086, 1.0180) | 0.0766 |
| Omega-3 fatty acids^6^, E% | 0.7751 (0.5075, 1.1840) | 0.2384 |
| Alpha linolenic acid, E% | 0.8986 (0.5895, 1.3700) | 0.6190 |
| Marine omega-3 fatty acids^3^, E% | 0.0761 (0.0120, 0.4845) | **0.0064** |
|  |  |  |
| Micronutrients |  |  |
| Calcium, mg | 0.9990 (0.9981, 0.9999) | **0.0234** |
| Folate, µg | 0.9942 (0.9907, 0.9978) | **0.0014** |
| Iodine, µg | 1.0030 (0.9991, 1.0070) | 0.1307 |
| Iron, mg | 1.0440 (0.9657, 1.1290) | 0.2780 |
| Magnesium, mg | 0.9951 (0.9912, 0.9990) | **0.0132** |
| Niacin, NE | 0.9693 (0.9260, 1.0150) | 0.1825 |
| Phosphorus, mg | 0.9980 (0.9969, 0.9992) | **0.0006** |
| Potassium, g | 0.9993 (0.9988, 0.9998) | **0.0048** |
| Riboflavin, mg | 1.1340 (0.7155, 1.7990) | 0.5916 |
| Selenium, µg | 0.9522 (0.9324, 0.9724) | **< 0.0001** |
| Sodium, mg | 1.0000 (0.9996, 1.0010) | 0.8705 |
| Thiamin, mg | 1.2070 (0.6399, 2.2750) | 0.5618 |
| Vitamin A, RE | 0.9980 (0.9968, 0.9993) | **0.0020** |
| Vitamin B12, µg | 0.9081 (0.7773, 1.0610) | 0.2246 |
| Vitamin B6, mg | 1.4710 (0.8537, 2.5330) | 0.1645 |
| Vitamin C, mg | 0.9989 (0.9933, 1.0050) | 0.7034 |
| Vitamin D, µg | 0.8714 (0.7832, 0.9695) | **0.0114** |
| Vitamin E, α-TE | 0.8236 (0.7337, 0.9245) | **0.0010** |
| Vitamin K, µg | 0.7767 (0.7276, 0.8292) | **< 0.0001** |
| Zinc, mg | 0.9495 (0.8243, 1.0940) | 0.4724 |
| β-carotene, µg | 0.9997 (0.9996, 0.9999) | **0.0001** |

Significant p-values marked in bold.

^1^ Patient and control modelled as dependent outcome, each nutrient as independent variable. Adjusted for previous smoking status (yes/no) country of birth (Sweden / abroad), residential status (single / shared living), educational level (1; junior high or missing data, 2; senior high school, 3; university or college), weight and energy intake. Variables expressed as intake per MJ or E% were not adjusted for energy intake.

^2^ Data missing from 12 controls.

^3^ The sum of EPA, DHA and DPA.

^4^ The sum of ALA and LA.

^5^ The sum of arachidonic acid and LA.

^6^ The sum of ALA, EPA, DHA and DPA.

Abbreviations: ALA, Alpha linoleic acid; DHA, Docosahexaenoic acid; DPA, Docosapentaenoic acid; EPA, Eicosapentaenoic acid; E%, Energy percentage; LA, Linoleic acid; MJ, megajoule; r-axSpA, radiographic axial SpondyloArthritis.

**Supplemental Table 2**. Patients with r-axSpA included in the comparison to controls, and patients not included

|  | **Total, n = 155** |  | **Included, n = 81** | | **Excluded, n = 74** | |  |
| --- | --- | --- | --- | --- | --- | --- | --- |
|  | Mean ± SD | Median (p25, p75) | Mean ± SD | Median (p25, p75) | Mean ± SD | Median (p25, p75) | P-value |
| Age (year) | 55.5 ± 11.4 | 57.2 (47.4, 64.6) | 57.9 ± 4.8 | 58.5 (53.8, 61.5) | 52.8 ± 15.4 | 46.7 (40.6, 69.1) | 0.082^1^ |
| BMI (kg/m^2^) | 27.9 ± 5.3 | 26.4 (24.4, 30.8) | 28.7 ± 6.0 | 26.9 (24.4, 32.4) | 27.0 ± 4.3 | 26.2 (24.4, 29.1) | **0.042**^2^ |
| Weight (kg) | 83.2 ± 19.1 | 80.8 (70.8, 91.9) | 84.9 (21.8) | 81.0 (70.8, 95.0) | 81.3 ± 15.5 | 77.9 (68.4, 90.7) | 0.2^2^ |
| Height (cm) | 172.4 ± 9.4 | 173.5 (165.2, 179.2) | 171.5 (9.6) | 171.8 (165.8, 178.5) | 173.3 ± 9.2 | 176.0 (164.9, 180.1) | 0.2^2^ |
| Age of diagnosis (year) | 32.3 ± 9.6 | 32.0 (25.0, 37.0) | 32.8 (8.6) | 32.0 (27.0, 37.5) | 31.8 ± 10.6 | 30.0 (24.8, 37.3) | 0.5^2^ |
| Symptom duration (year) | 31.8 ± 11.9 | 32.0 (24.0, 42.0) | 34.6 (8.4) | 34.0 (28.5, 42.0) | 28.8 ± 14.2 | 27.0 (16.0, 42.0) | **0.003**^2^ |
| Diagnosis duration (year) | 23.1 ± 11.3 | 23.0 (15.0, 31.0) | 25.1 (9.6) | 24.0 (17.5, 32.0) | 20.9 ± 12.5 | 21.0 (9.0, 30.3) | **0.023**^1^ |
| ASDAS-CRP (score) | 1.8 ± 0.7 | 1.8 (1.3, 2.2) | 1. 9 ± 0.8 | 1.8 (1.3, 2.3) | 1.7 ± 0.6 | 1.8 (1.2, 2.2) | 0.2^2^ |
| BASDAI^3^ (score) | 3.7 ± 1.9 | 3.5 (2.2, 5.3) | 4.0 ± 1.9 | 3.8 (2.3, 5.5) | 3.5 ± 1.8 | 3.5 (2.0, 5.0) | 0.1^2^ |
| BASFI (score) | 3.0 ± 2.0 | 2.6 (1.4, 4.2) | 3.3 ± 2.2 | 2.8 (1.5, 4.9) | 2.6 ± 1.9 | 2.3 (1.2, 4.1) | 0.090^1^ |
| mSASSS^4^ (score) | 18.9 ± 20.7 | 8.0 (1.0, 30.5) | 22.3 ± 22.1 | 16.0 (3.3, 36.3) | 13.2 ± 18.1 | 5.0 (0.0, 23.5) | **0.002**^1^ |
|  | N (%) |  | N (%) |  | N (%) |  | P-value^5^ |
| csDMARD and / or bDMARD | 38 (24.5) |  | 19 (23.5) |  | 19 (25.7) |  | 0.9 |
| csDMARD | 19 (12.3) |  | 9 (11.1) |  | 10 (13.5) |  | 0.8 |
| bDMARD | 27 (17.4) |  | 15 (18.5) |  | 12 (16.2) |  | 0.8 |
| NSAID |  |  |  |  |  |  | 0.9 |
| No | 26 (16.8) |  | 13 (16.0) |  | 13 (17.6) |  |  |
| On demand | 34 (21.9) |  | 19 (23.5) |  | 15 (20.3) |  |  |
| ≥ 2 days/week | 95 (61.3) |  | 49 (60.5) |  | 46 (62.2) |  |  |
| Male | 10 (6.5) |  | 54 (66.7) |  | 53 (71.6) |  | 0.6 |
| Single household | 34 (21.9) |  | 18 (22.2) |  | 16 (21.6) |  | 1.0 |
| Ever been smoker | 84 (54.2) |  | 39 (48.1) |  | 45 (60.8) |  | 0.1 |
| Educational level |  |  |  |  |  |  | **0.047** |
| Junior high school or  lower | 18 (11.6) |  | 6 (7.4) |  | 12 (16.2) |  |  |
| Senior high school | 84 (54.2) |  | 51 (63.0) |  | 33 (44.6) |  |  |
| College or university | 53 (34.2) |  | 24 (29.6) |  | 29 (39.2) |  |  |

Significant p-values marked in bold.

^1^ Mann Whitney test, using exact p-value

^2^ T-test (Student’s t-test or Welsh’s t-test depending on data variation).

^3^Data missing from one individual in the excluded group.

^4^ Data missing from two individuals, one from the included group and one in the excluded group.

^5^ Fisher’s exact test.

Abbreviations:

ASDAS-CRP, Ankylosing Spondylitis Disease Activity Score CRP; BASDAI, Bath Ankylosing Spondylitis Disease Activity Index; BASFI, Bath Ankylosing Spondylitis Functional Index; BMI, Body Mass Index; mSASSS, modified Stoke Ankylosing Spondylitis Spinal Score; csDMARD, conventional synthetic Disease Modifying Anti-Rheumatic Drug; bDMARD, biologic Disease-Modifying Anti-Rheumatic Drug; NSAID, Non-Steroidal Anti-Inflammatory Drug; r-axSpA, radiographic axial SpondyloArthritis.
